# Comparative Characterization of the Leaf Tissue of *Physalis alkekengi* and *Physalis peruviana* Using RNA-seq and Metabolite Profiling

**DOI:** 10.3389/fpls.2016.01883

**Published:** 2016-12-20

**Authors:** Atsushi Fukushima, Michimi Nakamura, Hideyuki Suzuki, Mami Yamazaki, Eva Knoch, Tetsuya Mori, Naoyuki Umemoto, Masaki Morita, Go Hirai, Mikiko Sodeoka, Kazuki Saito

**Affiliations:** ^1^RIKEN Center for Sustainable Resource ScienceYokohama, Japan; ^2^Graduate School of Pharmaceutical Sciences, Chiba UniversityChiba, Japan; ^3^Department of Biotechnology Research, Kazusa DNA Research InstituteChiba, Japan; ^4^Synthetic Organic Chemistry Laboratory, RIKENSaitama, Japan; ^5^RIKEN Center for Sustainable Resource ScienceSaitama, Japan

**Keywords:** *Physalis*, *de novo* transcriptome assembly, deep sequencing, marker development, secondary metabolism, physalin, withanolide

## Abstract

The genus *Physalis* in the *Solanaceae* family contains several species of benefit to humans. Examples include *P. alkekengi* (Chinese-lantern plant, hôzuki in Japanese) used for medicinal and for decorative purposes, and *P. peruviana*, also known as Cape gooseberry, which bears an edible, vitamin-rich fruit. Members of the *Physalis* genus are a valuable resource for phytochemicals needed for the development of medicines and functional foods. To fully utilize the potential of these phytochemicals we need to understand their biosynthesis, and for this we need genomic data, especially comprehensive transcriptome datasets for gene discovery. We report the *de novo* assembly of the transcriptome from leaves of *P. alkekengi* and *P. peruviana* using Illumina RNA-seq technologies. We identified 75,221 unigenes in *P. alkekengi* and 54,513 in *P. peruviana*. All unigenes were annotated with gene ontology (GO), Enzyme Commission (EC) numbers, and pathway information from the Kyoto Encyclopedia of Genes and Genomes (KEGG). We classified unigenes encoding enzyme candidates putatively involved in the secondary metabolism and identified more than one unigenes for each step in terpenoid backbone- and steroid biosynthesis in *P. alkekengi* and *P. peruviana*. To measure the variability of the withanolides including physalins and provide insights into their chemical diversity in *Physalis*, we also analyzed the metabolite content in leaves of *P. alkekengi* and *P. peruviana* at five different developmental stages by liquid chromatography-mass spectrometry. We discuss that comprehensive transcriptome approaches within a family can yield a clue for gene discovery in *Physalis* and provide insights into their complex chemical diversity. The transcriptome information we submit here will serve as an important public resource for further studies of the specialized metabolism of *Physalis* species.

## Introduction

The specialized or secondary metabolism in plants is an important source for fine chemicals including drugs, dyes, vitamins, and other chemical materials. The genus *Physalis*, the largest genera in the *Solanoideae* subfamily, contains the most economically important genera, e.g., *Solanum tuberosum* (potato), *S. lycopersicum* (tomato), and *Capsicum annuum* (red pepper; Martinez, 1998). Some plants in approximately 90 species have a long history of cultivation. *P. alkekengi var. franchetii* (Chinese-lantern, hôzuki in Japanese) has been used as a medicinal plant and for decorative purposes. *P. peruviana*, also known as Cape gooseberry, has an edible fruit that contains many vitamins and antioxidants (Ramadan and Morsel, 2003).

Members of the genus *Physalis* produce bioactive metabolites such as steroidal lactones withanolides (Eich, 2008). *P. peruviana* can produce withanolides and, *P. alkekengi* physalins, a different subgroup of withanolides. The structures of physalins A and B were first determined in 1969 (Matsuura and Kawai, 1969; Matsuura et al., 1969) and subsequently more than 30 physalins were isolated. Studies using spectroscopic methods isolated 3 new- and 7 known steroids including physalins and demonstrated that physalin B exhibited the most significant cytotoxic activities against HeLa human cervical cells (Kawai et al., 2002; Li et al., 2014). Physalin B or F inhibited NF-kappa B activation (Jacobo-Herrera et al., 2006; Wu et al., 2012) and both right- and left-sided partial structures were proposed to play a significant role in their mode of action (Ozawa et al., 2013). 4β-Hydroxywithanolide E derived from *P. peruviana* inhibited the growth of a human non-small lung cancer cell line (Yen et al., 2010).

High-throughput DNA sequencing with a next-generation sequencer (NGS) is useful for genome assembly, the detection of single nucleotide polymorphisms (SNPs) and genetic variations, and for transcriptome characterization (Wang et al., 2009; Garber et al., 2011). RNA sequencing (RNA-seq) on NGSs can be used for both model and non-model plants (Gongora-Castillo and Buell, 2013; Fukushima and Kusano, 2014; Weber, 2015). Successful transcriptome studies on non-model plants, e.g., crop plants such as the eggplant (Ramesh et al., 2016), pepper (Gordo et al., 2012) and tobacco (Lei et al., 2014) and medicinal plants, e.g., *Withania somnifera* (Gupta et al., 2013; Senthil et al., 2015) and *P. peruviana* (Garzon-Martinez et al., 2012) from the *Solanaceae* family have been published. Resources for transcriptome data from medicinal plants, e.g., the Medicinal Plant Genomics Resource (MPGR^1^) and the Medicinal Plants Transcriptomics (Medplants^2^; Gongora-Castillo et al., 2012a,b; Yeo et al., 2013) are available. To take full advantage of the potential of important phytochemicals we need to elucidate their biosynthesis. For this we need genomic data, especially comprehensive transcriptome datasets for gene discovery.

**FIGURE 1:**
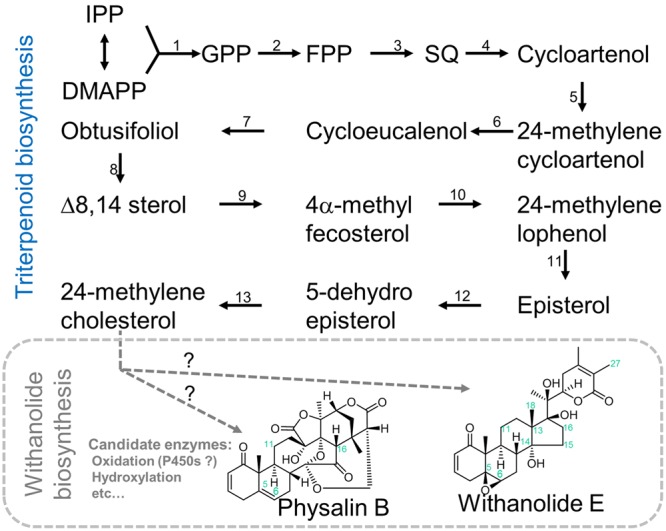
**Possible pathways of withanolide biosynthesis.** Our main aim of this study is to describe the whole transcriptome map of *P. alkekengi* and *P. peruviana* leaves and to inventory the basic information about a wide range of the metabolism including the withanolide biosynthesis pathway in leaf tissues. Although the assembled unigenes are hypothetic/proposed candidate genes, comparative transcriptome analysis using other *Physalis* species is a promising way to study and identify species-specific and evolutionary conserved pathways involved in highly complex biosynthesis of withanolides like physalin. Single black arrows show one step, two or more gray arrows show multiple unknown steps. Abbreviation: IPP, isopentenyl pyrophosphate; DMAPP, dimethylallyl pyrophosphate; GPP, geranyl pyrophosphate; FPP, farnesyl pyrophosphate; and SQ, squalene. Enzyme abbreviation: (1) Geranyl diphosphate Synthase (GPPS, EC 2.5.1.1); (2) Farnesyl diphosphate Synthase (FPPS, EC 2.5.1.10); (3), Squalene Synthase (SQS, EC 2.5.1.21); (4) Cycloartenol Synthase (CAS, EC 5.4.99.8); (5) Cycloartenol C-24 methyltransferase (SMT1, EC 2.1.1.41); (6) Sterol-4a-methyl oxidase (SMO, EC 1 1.14.13.72); (7) Cycloeucalenol cycloisomerase (CECI, EC 5.5.1.9); (8) obtusifoliol 14-demethylase (CYP51G1, EC 1.14.13.70); (9) D14-sterol reductase (FK, EC 1.3.1.70); (10) C-7,8 sterol isomerase (HYDI, EC 5.3.3.5); (11) Sterol-4a-methyl oxidase 2 (SMO2, EC 1.14.13.72); (12) C-5 sterol desaturase (STE1, EC 1.14.21.6); and (13) Sterol Δ7 reductase (DWF5, EC 1.3.1.21).

Our main aim of this study is to describe the whole transcriptome map of *P. alkekengi* and *P. peruviana* leaves and to inventory the basic information about a wide range of the metabolism including the withanolide biosynthesis pathway in leaves (**Figure 1**). Here we present a *de novo* assembly transcriptome from the leaves of *P. alkekengi* and *P. peruviana*. We employed Illumina RNA-seq techniques and demonstrate that these resources can provide the means for a better understanding of chemical diversity in *Physalis* species. We annotated the assembled unigenes in *P. alkekengi* and *P. peruviana* and classified unigenes encoding enzyme candidates putatively involved in secondary metabolism. Our approaches provide a basis for future researches on bio-engineering of *Physalis* plants and they represent a shortcut to gene discovery in these species.

## Materials and Methods

### Plant Materials, RNA Isolation, and cDNA Synthesis

*Physalis alkekengi var. franchetii* and *P. peruviana* (Ground Cherry or Golden Berry) plants were grown in the experimental gardens of the RIKEN Center for Sustainable Resource Science, Wako, Saitama, Japan. They are not an endangered or protected species. The third fresh leaves were collected from healthy plants (**Supplementary Image 1**). RNA isolation and cDNA synthesis for sequencing were carried out as previously reported (Fukushima et al., 2015; Han et al., 2015a,b). We used unreplicated data for one sample per species.

### Illumina Sequencing

For the generation of cDNA libraries we employed an Illumina HiSeq 2000 sequencer (Illumina Inc., San Diego, CA, USA). We sequenced 100-bp paired-end (PE) reads as described (Fukushima et al., 2015; Han et al., 2015a,b). Our short-read data in FASTQ file format were produced by Casava 1.8 (Illumina, Inc. San Diego, CA, USA). Short reads that did not pass Illumina’s standard quality filter were eliminated. The process yielded clean reads from the mRNA pool isolated from *P. alkekengi* and *P. peruviana*, respectively (**Table 1**).

**Table 1:** Summary of sequencing and assembly results after Illumina sequencing of *P. alkekengi* and *P. peruviana*.

Species	*P. alkekengi*	*P. peruviana*
Sequencing results		
Total length of clean reads (bp)	2,540,006,580	2,345,503,406
G + C%	43.1	43.4
Assembly results (unigenes)		
Number	75,221	54,513
Average length (bp)	867	930
Maximum length (bp)	14,564	12,329
Minimum length (bp)	201	201
N50 (bp)	1,550	1,636
G + C%	39.8	40.5

### Data Pre-processing and *De novo* Transcriptome Assembly

**FIGURE 2:**
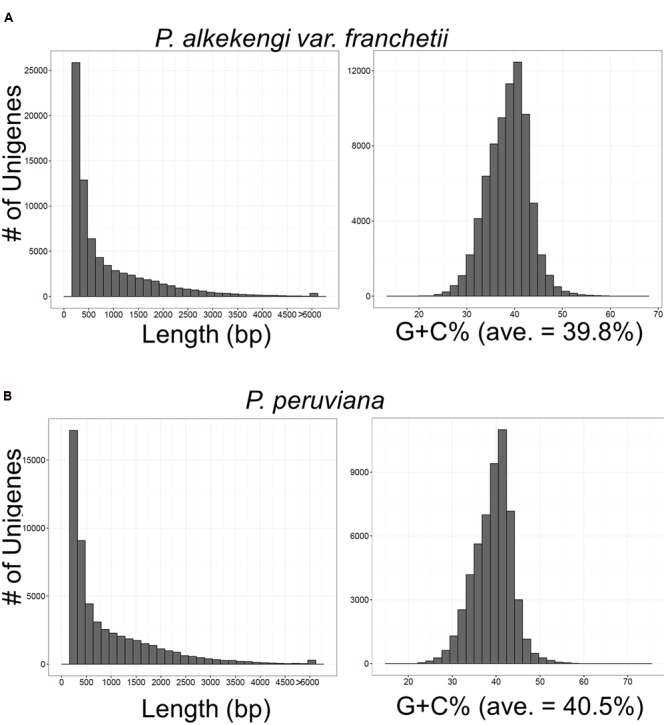
**Overview of the *de novo* transcriptome assembly in *P. alkekengi* (A)** and *P. peruviana* **(B)**. Length and G + C% distribution of unigenes assembled from high-quality clean reads by the Trinity program (Grabherr et al., 2011).

We performed *de novo* transcriptome assembly using the Trinity program (Grabherr et al., 2011). We then subjected the assembled unigenes to read alignment and transcript abundance estimation with Bowtie (Langmead et al., 2009) and RSEM (Li and Dewey, 2011). To estimate transcript abundance we used the Fragments Per Kilobase of exon per Million mapped fragments (FPKM) method. We identified 75,221 unigenes in *P. alkekengi* and 54,513 unigenes in *P. peruviana*. The length and G + C% distribution of all unigenes are shown in **Figures 2A,B**. The G + C% and basic statistics were calculated with the custom Ruby/Bioruby script (Goto et al., 2010), “Biostrings” (Pages et al., 2009), and the R/Bioconductor package “ShortRead” (Morgan et al., 2009). For quality control we used the FASTX-Toolkit ^3^ and the FastQC package^4^. Default settings were used in all calculations in this section.

### Functional Annotation and Classification of Unigenes

We performed functional annotation of all unigenes with a BLASTx search (Altschul et al., 1990) against the NCBI NR database^5^ (formatted on April 7, 2014); our cutoff *E*-value was <1*E*-5. We applied the Blast2GO program v 2.7.1 (Conesa et al., 2005) to assign GO categories, an EC number, and KEGG pathways (Kanehisa et al., 2014) based on the BLAST results with default settings. Visualization of the GO functional category of all unigenes and of the distribution of gene functions in the different species was with the BGI WEGO program (Ye et al., 2006). We identified microsatellites in the unigenes using the microsatellite identification tool (MISA)^6^ (Thiel et al., 2003). The default parameters (unit size-minimum repeats: 1-10, 2-6, 3-5, 4-5, 5-5, and 6-5) were used.

### Authentic Samples and Extraction of Withanolides and LC-QTOF-MS Analysis

The authentic samples of withanolides were isolated from plants as described in our previous paper (Ozawa et al., 2013). The leaves of *P. alkekengi* and *P. peruviana* were collected at five different developmental stages of the growing season before bearing fruit, because our pilot study showed to detect withanolides of *P. alkekengi* and *P. peruviana* at these stages (data not shown). Fresh samples of leaves at five different developmental stages were extracted with 5 μl of 80% MeOH containing 2.5 μM lidocaine (internal standard) per mg fresh weight using a mixer mill with zirconia beads (7 min at 18 Hz and 4°C). After 10-min centrifugation, the supernatant was filtered using an HLB μElution plate (Waters). The extracts (1 μl) were analyzed with LC-QTOF-MS (LC, Waters Acquity UPLC system; MS, Waters Xevo G2 Q-Tof). The analytical conditions for metabolite profiling were as described in elsewhere (Tamura et al., 2014). The polarity of electrospray ionization was applied in positive ionization mode.

## Results and Discussion

### Sample Preparation, Illumina Sequencing, and *De novo* Transcriptome Assembly

For the transcriptome analysis of *P. alkekengi* and *P. peruviana*, total RNA samples were isolated from leaves (**Supplementary Image 1**). We performed DNase treatment and confirmed RNA integrity using a bioanalyzer (see Materials and Methods). Total RNA was used in mRNA preparation, fragmentation, and cDNA synthesis. Illumina sequencing produced clean reads (in total 2,540,006,580 bp and 2,345,503,406 bp) from the mRNA pool isolated from *P. alkekengi* and *P. peruviana*, respectively (**Table 1**). The short reads exhibited mean quality scores of 35.0 in *P. alkekengi* and 34.9 in *P. peruviana*, confirming that our sequencing was sufficient for *de novo* assembly. After the removal of adaptor-, ambiguous- and low-quality reads, we assembled all clean reads into unigenes using the Trinity program (Grabherr et al., 2011). There were 75,221 and 54,513 total transcripts in leaves of *P. alkekengi* and *P. peruviana*, respectively. The unigenes in *P. alkekengi* had an average length of 867 basepairs (bp) and an N50 of 1,550 bp; in *P. peruviana* the average length was 930 and 1,636 bp (N50). The length and the G + C% distribution for all unigenes in *P. alkekengi* and *P. peruviana* are shown in **Figure 2**. In total, 39,709 unigenes were less than 500 bp in length. In *P. alkekengi* 2,732 unigenes were longer than 3,000 bp. In *P. peruviana* 26,991 unigenes were less than 500 bp and 2,283 were longer than 3,000 bp (Supplementary Data Sheet 1). The average G + C content of unigenes in *P. alkekengi* and *P. peruviana* was 39.8 and 40.5%, respectively. The G + C content in *P. peruviana* is consistent with what has been reported previously (Garzon-Martinez et al., 2012).

### Functional Annotation of *Physalis* Unigenes

**FIGURE 3:**
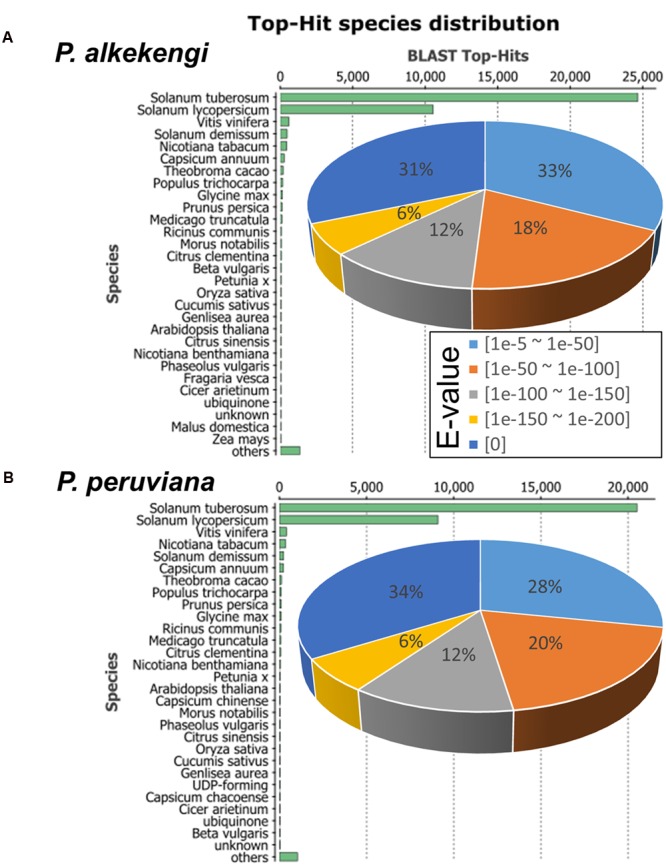
**Characterization of the assembled unigenes based on a non-redundant (NR) protein database search in *Physalis*. (A)** Top-hit species- and *E*-value-distributions of *P. alkekengi*. The *E*-value distribution of BLAST hits for the assembled unigenes with a cutoff of *E*-value < 1*E*-5. **(B)** Top-hit species- and *E*-value-distributions of *P. peruviana*. Top-hit distribution was calculated based on only the best/top sequence alignment with the lowest *E*-value for our BLAST result.

To assess and annotate the assembled unigenes, we performed a sequence homology search against the GenBank non-redundant (NR) database using BLASTx (*E*-value < 1*E*-5; Altschul et al., 1990). We found that 40,090 and 33,183 unigene sequences in *P. alkekengi* and *P. peruviana*, respectively, had BLAST hits to annotated sequences in the NR database (**Figure 3**). Further analysis of the similarity distributions showed that 72.5 and 74.7% of matched sequences in *P. alkekengi* and *P. peruviana*, respectively, manifested alignment identities greater than 80%. For *P. alkekengi* a large number of the best/top-hits matched the sequences of *S. tuberosum* (61.5%) and *S. lycopersicum* (26.3%); other hits were detected within the reference protein databases of *Vitis vinifera* (1.5%) and *S. demissum* (1.2%; **Figure 3A**). The *P. peruviana* transcriptome showed that the hits matched the sequences of *S. tuberosum* (61.8%) and *S. lycopersicum* (27.4%); other hits were detected within the reference protein databases of *V. vinifera* (1.2%) and *Nicotiana tabacum* (1.0%; **Figure 3B**). These observations indicate that the distribution of the top BLAST hits for the obtained unigenes from two different *Physalis* species was similar. Unigenes with no BLAST hits may imply the presence of additional genes that do not exist in the annotated sequence databases or of sequences too short for BLAST hits.

**FIGURE 4:**
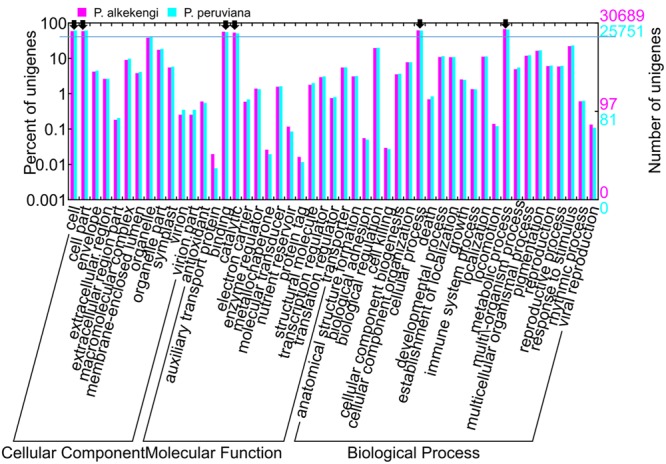
**GO assignments for all assembled unigenes in *P. alkekengi* and *P. peruviana*.** The results are summarized in sets of three functional categories: cellular component, molecular function, and biological process. 30,689 and 25,751 unigenes from *P. alkekengi* (magenta) and *P. peruviana* (cyan), respectively, were categorized by GO categories. The GO categories were displayed using WEGO (http://wego.genomics.org.cn; Ye et al., 2006).

The gene ontology (GO), classification of standardized gene functions, is useful for annotating gene functions and gene products in any organism. GO contains three main independent categories: cellular component, molecular function, and biological process (The Gene Ontology Consortium, 2014). We used Blast2GO software (Conesa et al., 2005) to analyze the GO functional categories of the assembled unigenes and then applied WEGO program (Ye et al., 2006) to visualize the results of GO functional classifications. WEGO maps all annotated unigenes to GO categories and detect the number of unigenes associated with each GO category. Based on NR annotation, 30,689 and 25,751 unigenes in *P. alkekengi* and *P. peruviana*, respectively, were assigned to three main categories (**Figure 4**). Further analysis of the GO categories showed that the dominant categories were “cell,” “cell part,” “binding,” “catalytic,” “metabolic process,” and “cellular process” (black arrows in **Figure 4**). We observed that within the biological process group, most unigenes were putatively involved in “cellular process” and “metabolic process.” Most unigenes were assigned to the GO categories “binding” and “catalytic activity” in the molecular function group, and to “cells” and “cell parts” in the cellular component. Please note that these observation depends on the layer of the GO categories.

**FIGURE 5:**
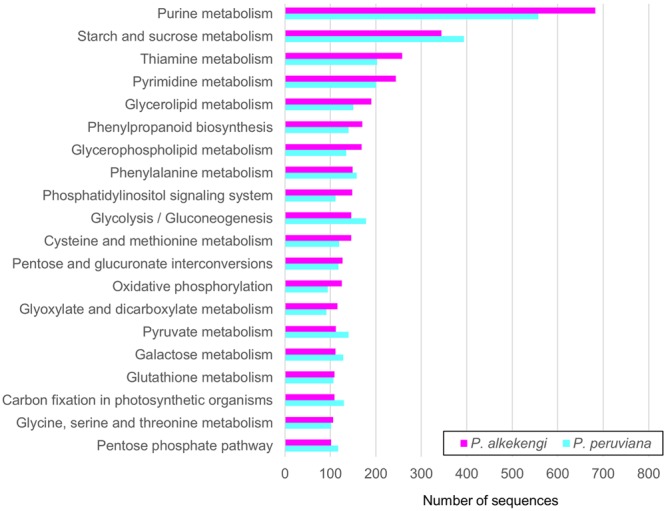
**Pathway enrichment analysis of assembled unigenes in *Physalis*.** Annotated unigenes were classified into 134 and 139 KEGG pathways in *P. alkekengi* and *P. peruviana*, respectively. The top 20 pathways including unigenes in *P. alkekengi* and the corresponding pathways in *P. peruviana* are displayed.

KEGG (Kanehisa et al., 2014) is a database resource for various levels of molecules in biological systems. It provides useful information for exploring the functional characteristics of genes. To further elucidate the function of the *Physalis* transcriptomes, the unigenes were annotated by the KEGG pathway. A total of 3,819 and 3,669 unigenes in *P. alkekengi* and *P. peruviana* was assigned into 134 and 139 KEGG pathways, respectively (**Figure 5** and Supplementary Data Sheet 2). The top-five ranking pathways in *P. alkekengi* were purine- (683 unigenes), starch and sucrose- (344 unigenes), thiamine- (258 unigenes), pyrimidine- (244 unigenes), and glycerolipid metabolism (190 unigenes). The top-five ranking pathways in *P. peruviana* were similar to *P. alkekengi* except for glycolysis/gluconeogenesis (178 unigenes in *P. peruviana*; Supplementary Data Sheet 2). These results agree with a previous study that performed the *P. peruviana* leaf transcriptome (Garzon-Martinez et al., 2012). In addition, we observed a higher number of the classified unigenes putatively involved in starch and sucrose- and glycolysis metabolism in *P. peruviana* than that of *P. alkekengi*. Such gene diversity derived from genomic architecture may reflect different source-to-sink balance between the both species, causing different photosynthetic rate and the carbon partitioning and allocation [for example, see (Osorio et al., 2014)]. This analysis implies that the pathway-based approach (see reviews by Ramanan et al., 2012; Fukushima et al., 2014) is useful for a better understanding of biological functions, gene interactions, and specific processes in *Physalis* species.

### Candidate Gene Families Associated with Withanolide Biosynthesis

To visualize diversity in secondary metabolism, we classified unigenes into gene families (Supplementary Data Sheet 2C). The resultant table shows unigenes encoding enzyme candidates putatively involved in the biosynthesis of secondary metabolites such as alkaloids, terpenoids, steroids, and flavonoids. For terpenoid backbone- and steroid biosynthesis we identified more than one unigene for each step in *P. alkekengi* and *P. peruviana*. For example, unigenes encoding enzyme candidates putatively involved in the biosynthesis of 24-methylene cholesterol from isopentenyl pyrophosphate (IPP) and dimethylallyl pyrophosphate (DMAPP) [the synthesis requires 13 reaction steps (Gupta et al., 2013, 2015)] contained Δ7 desaturase (EC:1.14.21.6), 24-C-methyltrasferase (EC:2.1.1.41), squalene epoxidase (EC: 1.14.13.132→1.14.14.17), squalene synthase (EC: 2.5.1.21), cholestenol delta-isomerase (EC: 5.3.3.5), and sterol Δ7-reductase (EC:1.3.1.21). Sterol Δ7-reductase catalyzes the biosynthesis of 24-methylene cholesterol, a central precursor in withanolide biosynthesis (Lockley et al., 1976). Gupta et al. demonstrated that silencing of sterol Δ7-reductase in *W. somnifera* elicited a reduction in the level of the major withanolide withaferin A in leaves (Gupta et al., 2015).

**FIGURE 6:**
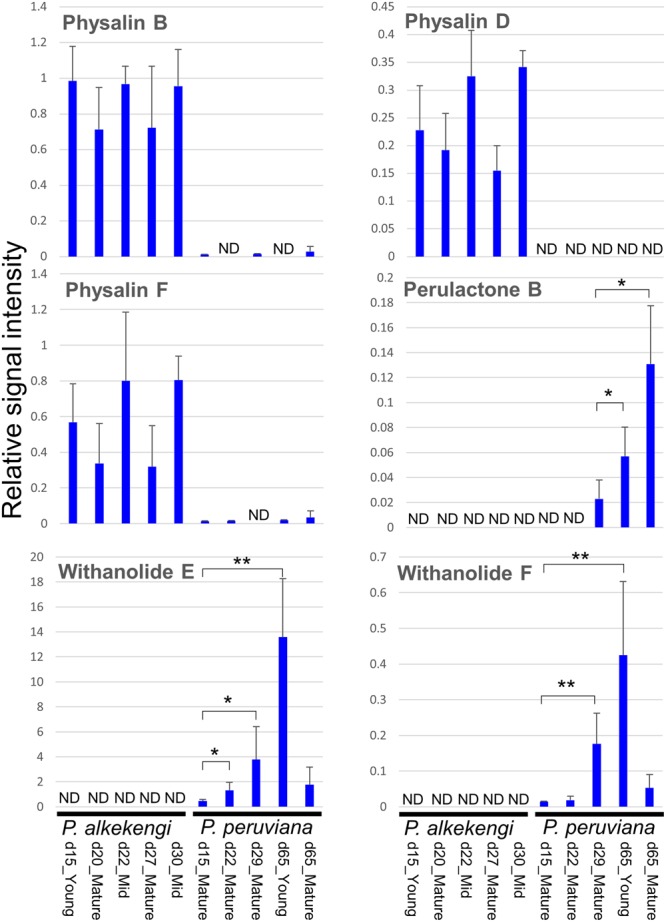
**Measurement of six withanolides of the leaves from *P. alkekengi* and *P. peruviana*.** Different developmental stages and leaf ages were used for comparison between *P. alkekengi* and *P. peruviana*. The analysis was performed with 3–6 biological replicates for each tissue/leaf age. An error bar indicates standard deviation. The authentic compounds used in this analysis were chemically synthesized as described in our previous paper (Ozawa et al., 2013). The limit of detection of each sample was 0.011923 (*P. alkekengi*, d15_Young), 0.010910 (*P. alkekengi*, d20_Mature), 0.013556 (*P. alkekengi*, d22_Mid), 0.010820 (*P. alkekengi*, d27_Mature), 0.010884 (*P. alkekengi*, d30_Mid), 0.012211 (*P. peruviana*, d15_Mature), 0.012456 (*P. peruviana*, d22_Mature), 0.012366 (*P. peruviana*, d29_Mature), 0.014794 (*P. peruviana*, d65_Young), and 0.013141 (*P. peruviana*, d65_Mature). Asterisks represent statistically significant differences from the sample of the youngest stage (i.e., d29_Mature for perulactone B and d15_Mature for withanolide E and F; *t*-test, ^∗^ *p* < 0.05 and ^∗∗^ *p* < 0.01). Abbreviation: d, day; Mid, middle; and ND, not detected.

The specific withanolide contents in leaves of *P. alkekengi* and *P. peruviana* at different stages of the leaf development remain largely unknown. To monitor the variability of the withanolides including physalins and provide insights into the chemical diversity of *Physalis*, we analyzed the metabolite content in leaves of *P. alkekengi* and *P. peruviana* at five different developmental stages by liquid chromatography-quadrupole time-of-flight-mass spectrometry (LC-QTOF-MS), focusing on six withanolide metabolites, i.e., physalin B, D, F, withanolide E and F, and perulactone B (**Supplementary Image 2**). We found that *P. alkekengi* produced high levels of physalin B, D, and F. In *P. peruviana* we only detected withanolide E and F, and perulactone B (**Figure 6**). Low levels of withanolide E, F, and perulactone B in *P. peruviana* were observed at the early developmental stages, while the highest levels of withanolide E and F were detected in the young leaves at day 65 (*t*-test, *p* < 0.01). A possible explanation for the difference in metabolite levels could be that there are sequence diversity in candidate genes putatively involved in various secondary transformations, including hydroxylation, glycosylation, methylation, and oxidation/reduction, to produce species-specific and development-specific withanolides from 24-methylene cholesterol as a substrate.

To further narrow down the candidate genes responsible for the biosynthesis of withanolides, we sought to identify unigenes that are uniquely expressed in either species. Such unigenes may play important roles in the difference of metabolic phenotypes. To this end we performed reciprocal best-hit BLAST search to identify homologous unigenes in *P. alkekengi* and *P. peruviana* with BLASTn (*E*-value < 1*E*-14) resulting in 25,670 homologous unigenes. Of these unigenes, we identified unigenes expressed uniquely in either of these species (Supplementary Data Sheet 3). We uncovered 234 unigenes that are exclusive to *P. alkekengi* (FPKM > 1). Of these, a cytochrome p450 chloroplastic-like protein (unigene-ID: c13295_g2_i2) and oxidoreductase-like protein (c16207_g5_i1), are possible candidate genes for the oxidations at the C-15 and C-18 positions of the steroid backbone required in the synthesis of physalis (**Figure 1**). In contrast, 355 unigenes found in *P. peruviana* were identified through this approach. There were unigenes encoding sterol reductase (c27112_g1_i1), methyltransferase family proteins, and transcription factors like MYB as candidates of specialized metabolites that are likely produced in *P. peruviana*. We had expected some obvious, differentially expressed candidates from the comparative transcriptomics, as was reported by (Yamazaki et al., 2008). However, the list of genes expressed exclusively in either *P. alkekengi* or *P. peruviana* provided only few candidates. The transcriptomes of both *P. alkekengi* and *P. peruviana* contain many cytochrome p450 monooxygenases and dioxygenases that could be modify both withanolides and physalins. However, none of these stand out as predominant in either *Physalis* species. We expect the biosynthesis of physalins to include cleavage of the C13-C14 bond as well as crosslinking between C14 and C27. Currently we have no good candidate enzymes for these reactions. Cleavage of the C13-C14 bond may occur through Grob fragmentation by an oxidosqualene cyclase type enzyme as described by Xiong et al. (2006).

Although there was no convincing evidence for significant changes in gene expressions encoding any of the key enzymes, we were able to provide a set of the hypothetical candidate genes involved in the pathways. We also recognize the limitation of our metabolite analysis in all its quantitative aspects, and that further assessment of the integration of our transcriptome data with wider metabolite profile is needed, but the findings presented here can provide a relevant resource for future study in *Physalis*. Our transcriptome dataset can be used for the identification of genes that contribute to the diversification in *Physalis* species-specific secondary metabolism, such as glycosyltransferases, methyltransferases, and cytochrome P450s.

### Identification and Comparison of Single Sequence Repeat (SSR) Markers

Single sequence repeats SSRs are microsatellites, repetitive DNA sequences in eukaryotes. They are useful markers in population genetics research, genetic map construction, and genetic diversity assessment (for example, see reviews Hancock, 1996; Varshney et al., 2005). To detect and compare SSRs in the two different *Physalis* species we performed *in silico* SSR marker identification with MISA (Thiel et al., 2003). We identified 11,415 SSRs in 75,221 transcripts of *P. alkekengi* and 12,180 SSRs in *P. peruviana* (**Table 2** and Supplementary Data Sheet 4). All SSRs can be classified by their repeat-unit sizes. Mono-nucleotide SSRs represented the largest portion (60.9%) of identified SSRs, followed by tri-nucleotide- (19.8%) and di-nucleotide (18.2%) SSRs. Although only a small portion of tetra- (*n* = 86), penta- (*n* = 14), and hexa-nucleotide SSRs (*n* = 19) were detected in *P. alkekengi* transcripts, it was significant. *P. peruviana* sequences showed nearly the same tendency and had more SSRs than *P. alkekengi*, especially mono- and tri-nucleotide SSRs. There were small numbers of tetra-, penta- and hexa-nucleotide SSRs in our transcriptome sequences from the two species, implying that their base patterns were highly complex. The SSRs of both *P. alkekengi* and *P. peruviana* may yield potential genetic markers for a wide range of investigations including population genetics-, comparative genomic-, and gene-based association studies aimed at elucidating the genetic control of important traits.

**Table 2:** Statistics of SSRs detected in *P. alkekengi* and *P. peruviana*.

Summary of microsatellite search	*P. alkekengi*	*P. peruviana*
Total number of identified SSRs	11,415	12,180
Number of SSR-containing unigenes	9,565	9,520
Number of unigenes containing more than 1 SSR	1,464	2,013
Number of SSRs present in compound formation	756	1,025
*Distribution to different type of repeats*		
*# of SSRs (unit size = 1)*	6,955	7,364
2	2,078	1,980
3	2,263	2,721
4	86	83
5	14	14
6	19	18

## Conclusion

We present the comprehensive transcriptome of the leaves of *P. alkekengi* and *P. peruviana* and provide a large-scale resource for assembled and functionally annotated gene candidates. Deep transcriptome analysis provided 75,221 unigenes in *P. alkekengi* and 54,513 in *P. peruviana*, respectively. A BLAST search of these sequences identified 40,090 and 33,183 unigenes in *P. alkekengi* and *P. peruviana* as annotated proteins, respectively. We discussed our findings on the gene candidates putatively involved in withanolide biosynthesis and their transcript- and metabolite profiles. Our detection of 11,415 SSRs in *P. alkekengi* and 12,180 SSRs in *P. peruviana* provides a useful resource for population genetics studies, genetic map construction, and genetic diversity assessment in *Physalis*. Because the *Physalis* genus is valuable for producing medicines and functional foods, more genomic data on other members are needed. Our study suggests that comprehensive approaches applied within the family can provide a clue to gene discovery in *Physalis* and yield insights into their complex diversity. Given the insufficient knowledge on the molecular mechanisms controlling the biosynthetic pathways involved in various bioactive metabolites like withanolides, the transcriptome information reported here represents an important public resource for further study on the specialized metabolism of *Physalis* species.

## Data Accessibility

All raw read sequences in FASTQ format can be downloaded from the DDBJ Sequence Read Archive (Kosuge et al., 2014) under accession number DRA004085. Our assembled transcripts were deposited as a Transcriptome Shotgun Assembly (TSA) in GenBank/EMBL/DDBJ under the accession IABG01000001-IABG01075221 (75221 entries for *P. alkekengi*) and IABH01000001-IABH01054513 (54513 entries for *P. peruviana*).

## Author Contributions

Conceived and designed the experiments: AF, HS, MY, MS, and KS. Performed the experiments: MN, HS, MY, TM, MM, GH, and MS. Analyzed the data: AF, EK, NU, TM, and HS. Contributed reagents/materials/analysis tools: MN, HS, MY, TM, MM, GH, and MS. Wrote the paper: AF, EK, and KS. All authors read and approved the final manuscript.

## Conflict of Interest Statement

The authors declare that the research was conducted in the absence of any commercial or financial relationships that could be construed as a potential conflict of interest.

## Footnotes

1 http://medicinalplantgenomics.msu.edu/

2 http://medplants.ncgr.org/

3 http://hannonlab.cshl.edu/fastx_toolkit/

4 http://www.bioinformatics.babraham.ac.uk/projects/fastqc/

5 http://www.ncbi.nlm.nih.gov

6 http://pgrc.ipk-gatersleben.de/misa/

## Supplementary Material

IMAGE 1 **Schematic workflow of this study.** We identified 75,221 and 54,513 transcripts in leaves of *P. alkekengi* and *P. peruviana* with the Trinity program (Grabherr et al., 2011). Assembled unigenes were annotated by Blast2GO program (Conesa et al., 2005).

IMAGE 2 **Structures of complex and oxidized steroidal constituents isolated from *Physalis* plants.** We analyzed the metabolite content in leaf tissues of *P. alkekengi* and *P. peruviana* at five different developmental stages by liquid chromatography-quadrupole time-of-flight-mass spectrometry (LC-QTOF-MS), focusing on these six withanolide metabolites.
