# Supplementary figures and images for: Comparative Characterization of the Leaf Tissue of Physalis alkekengi and Physalis peruviana Using RNA-seq and Metabolite Profiling
Source: Front Plant Sci. 2016 Dec 20;7:1883. doi: 10.3389/fpls.2016.01883 (PMC5167740; doi:10.3389/fpls.2016.01883)

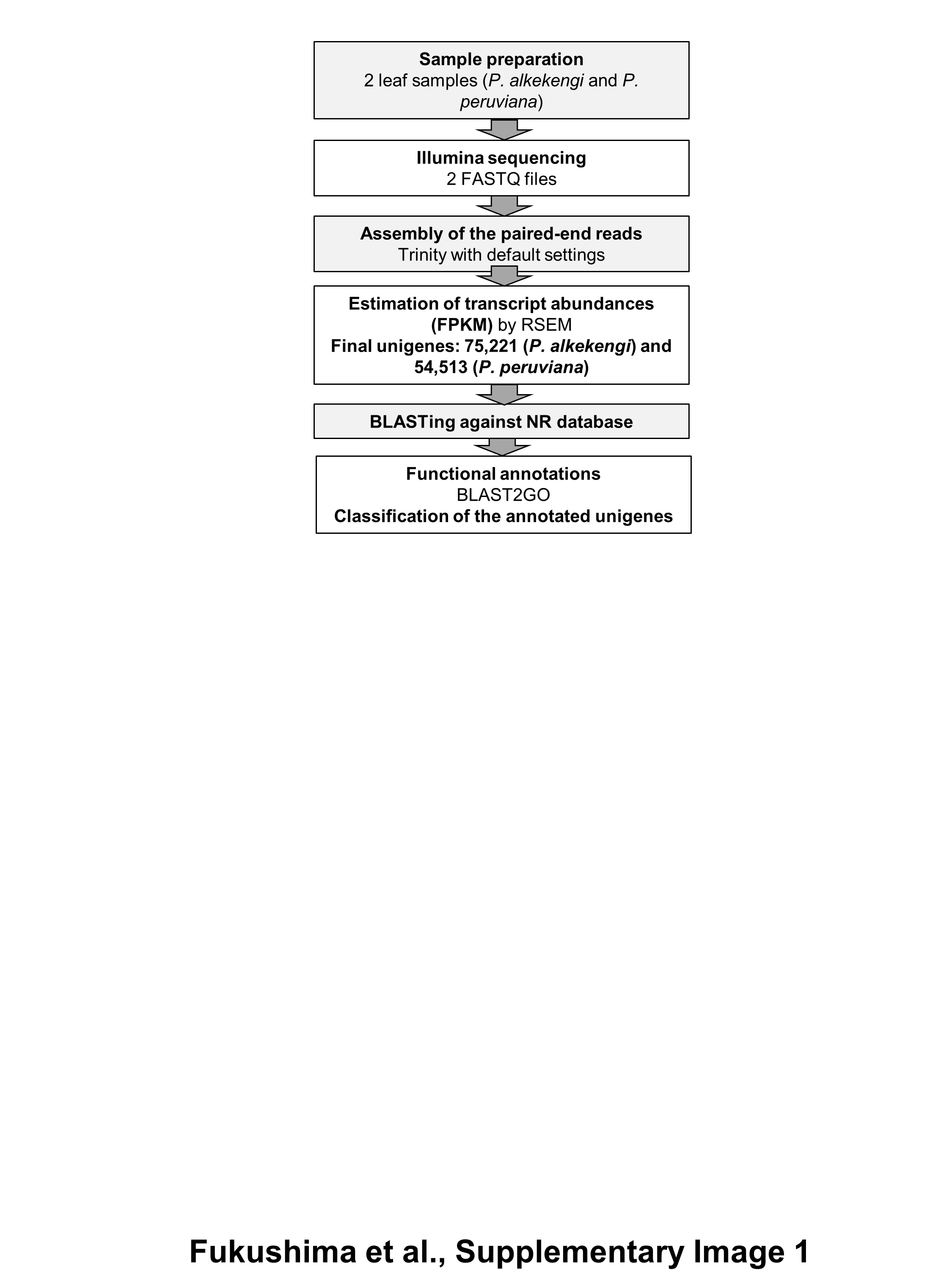

Supplement: IMAGE 1 — Schematic workflow of this study. We identified 75,221 and 54,513 transcripts in leaves of P. alkekengi and P. peruviana with the Trinity program (Grabherr et al., 2011). Assembled unigenes were annotated by Blast2GO program (Conesa et al., 2005). [file Image_1.TIF]

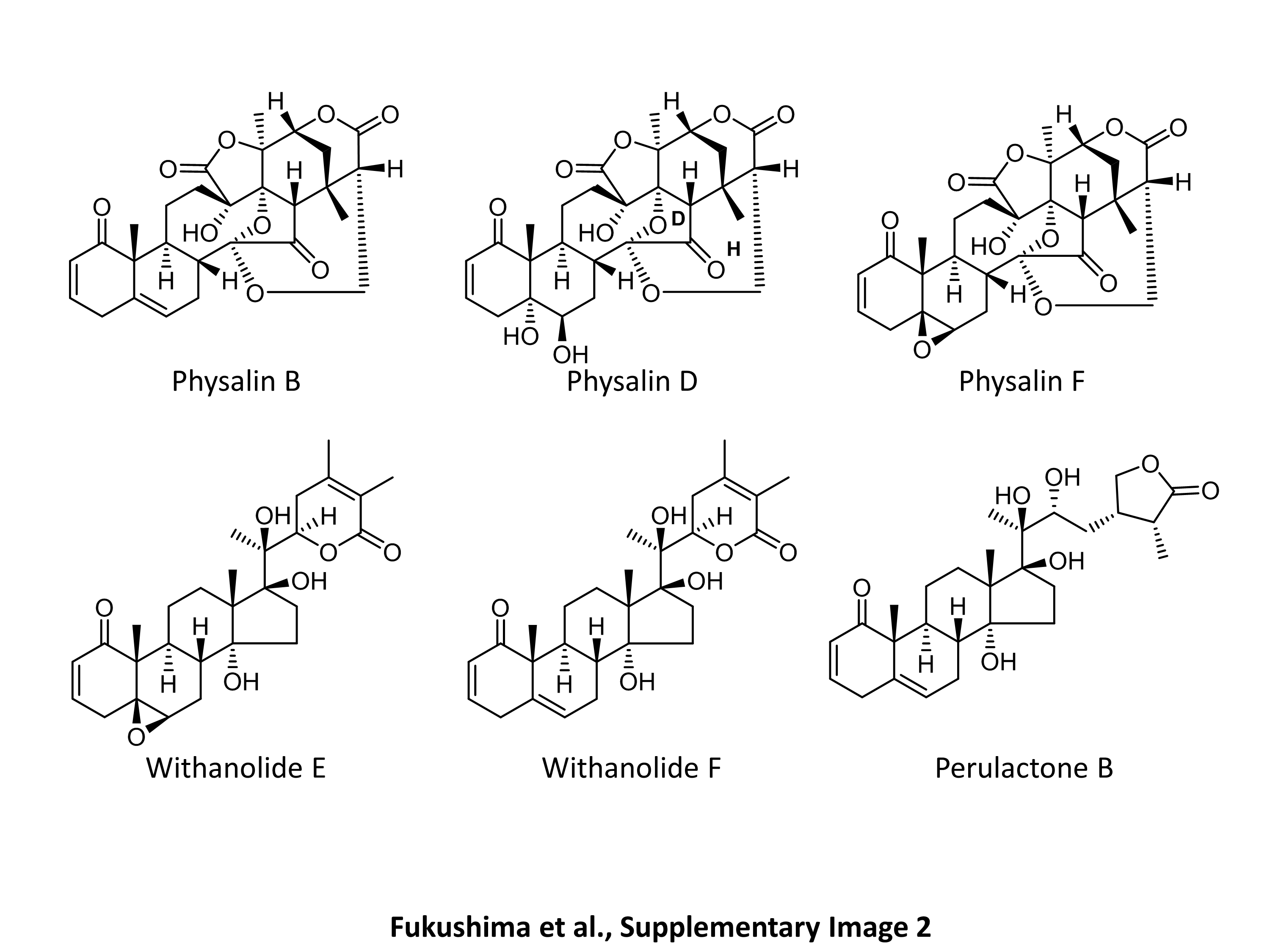

Supplement: IMAGE 2 — Structures of complex and oxidized steroidal constituents isolated from Physalis plants. We analyzed the metabolite content in leaf tissues of P. alkekengi and P. peruviana at five different developmental stages by liquid chromatography-quadrupole time-of-flight-mass spectrometry (LC-QTOF-MS), focusing on these six withanolide metabolites. [file Image_2.TIF]
